# Efficacy and Metabolic Effect on Serum Lipids of Apremilast in Psoriatic Arthritis: A Case Report

**DOI:** 10.3390/jcm8030398

**Published:** 2019-03-22

**Authors:** Roberta Gualtierotti, Orazio De Lucia

**Affiliations:** 1 Dipartimento di Scienze Cliniche e di Comunità, Università degli Studi di Milano, 20122 Milano, Italy; 2 Dipartimento di Reumatologia e Scienze Mediche, ASST Centro Specialistico Ortopedico Traumatologico Gaetano Pini-CTO, 20122 Milano, Italy; orazio.delucia@asst-pini-cto.it

**Keywords:** psoriatic arthritis, psoriasis, apremilast, lipid profile, DMARD

## Abstract

Psoriatic arthritis (PsA) is a chronic immune-mediated disease manifesting as joint inflammation with functional impairment associated with psoriasis. Recently, PsA has emerged as a systemic disease with several comorbidities, such as cardiovascular diseases and metabolic disorders. Apremilast is a targeted synthetic disease-modifying anti-rheumatic drug (tsDMARD) directed against phosphodiesterase 4 (PDE4) with demonstrated efficacy and safety in PsA and psoriasis. We report the case of a patient with PsA manifesting as arthritis, dactylitis, mild psoriasis and a significantly reduced health-related quality of life (HRQoL). Treatment with apremilast in association with methotrexate led to a quick improvement of joint and skin involvement with a stable amelioration of HRQoL. Furthermore, we observed a persistent favorable shift of serum lipid profile. Our observations suggest that apremilast is effective in controlling mild skin and joint involvement, including dactylitis, and suggest a potentially advantageous metabolic effect in patients with PsA.

## 1. Introduction

Psoriatic arthritis (PsA) is a chronic immune-mediated inflammatory arthropathy associated with psoriasis. It is characterized by multiple manifestations such as peripheral and/or axial arthritis, enthesitis, dactylitis, psoriasis, and nail involvement. In recent decades it has become clear that PsA is a systemic disease associated with several co-morbidities, such as cardiovascular (CV) diseases and metabolic disorders, that overall negatively impact survival. Furthermore, PsA often affects patients’ health-related quality of life (HRQoL) [1,2]. 

Despite the advances in the treatment of PsA with conventional synthetic (cs) disease-modifying anti-rheumatic drugs (DMARDs) and biological (b) DMARDs, inadequate response, drug intolerance and therapeutic resistance have led to the development of new molecules such as apremilast, a targeted synthetic (ts) DMARD [3]. Apremilast is a phosphodiesterase 4 (PDE4) inhibitor approved for the treatment of PsA as a second-line agent after failure of csDMARDs and a contraindication to bDMARDs, particularly if the patient presents peripheral arthritis, enthesitis or dactylitis [4,5,6]. Apremilast has demonstrated effectiveness in the treatment of mild-moderate PsA, a favorable safety profile and emerging advantageous metabolic effects in randomized controlled trials (RCTs) [7,8,9]. However, to date, there have been few real-life reports. 

We here report the case of a patient with PsA with mild skin and joint involvement safely treated with apremilast in association with methotrexate, leading to a quick clinical response, a considerable amelioration of HRQoL and a favorable serum lipid profile. Our observations are relevant because they support the efficacy of apremilast in the treatment of mild-moderate psoriasis and PsA and report for the first time a possible metabolic effect on serum lipid profile. This case report has been written and edited following the case report (CARE) writing guidelines [10].

## 2. Case Report

**Figure 1:**
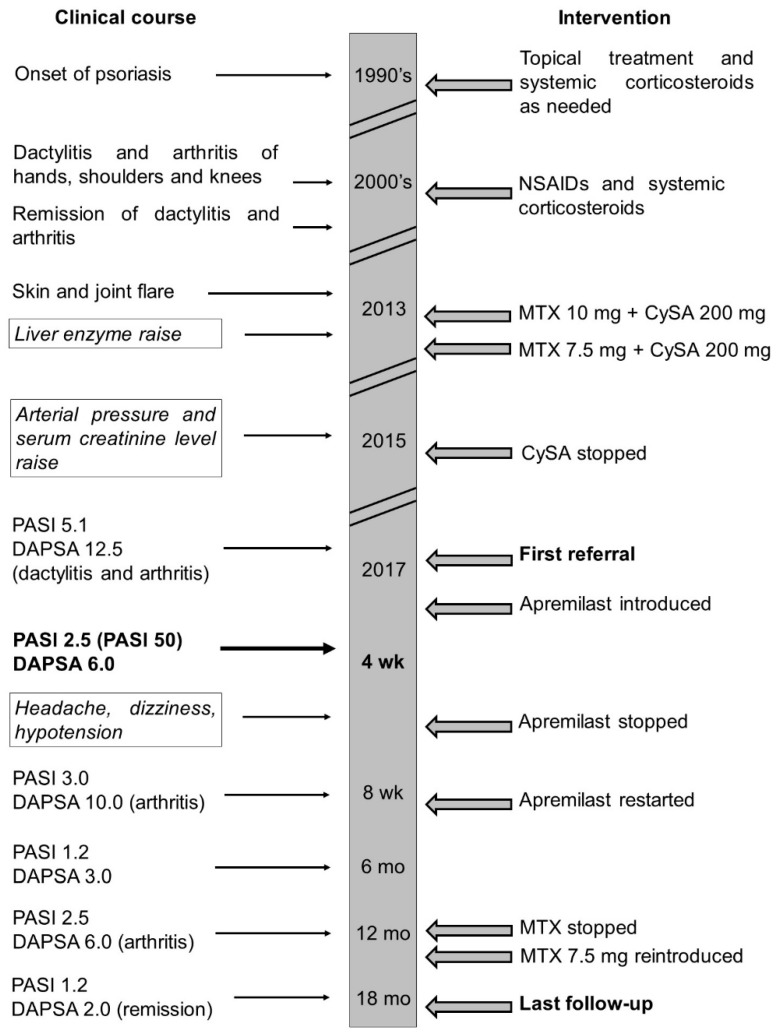
Case report timeline. Clinical course (**left**) and interventions (**right**) are described. Adverse effects are framed and written in italics. The first referral, the last follow-up visit, and the 4 week follow-up examination are reported in bold. Definitions: cyclosporine A (CySA); non-steroidal anti-inflammatory drugs (NSAIDs); methotrexate (MTX); psoriasis area severity index (PASI); disease activity in psoriatic arthritis (DAPSA); weeks (wk); months (mo).

We present the case of a 45-year-old Caucasian man suffering from psoriasis for more than 20 years at the time of the first visit. No relevant comorbidities were found at history collection, apart from benign prostatic hyperplasia, for which he is treated with tamsulosine. He was initially diagnosed and followed-up by dermatologists, who treated him with topical corticosteroids and salicylic acid for skin involvement and systemic corticosteroids for resistant lesions as needed (Figure 1). 

The skin component had always been <30% of the total body surface area (BSA). The patient reported distress in personal and social relationships with consequently reduced HRQoL. After ten years, he started experiencing dactylitis of feet and recurrent arthritis of the small joints of hands, shoulders and knees, together with inflammatory morning stiffness lasting around one hour, with negative rheumatoid factor (RF); thus fulfilling ClASsification criteria for Psoriatic ARthritis CASPAR criteria for PsA [11]. He was referred to the rheumatology outpatient clinic of another hospital and treated with indomethacine and systemic corticosteroids as needed for about five years, and then achieved complete remission of joint symptoms. Three years later, because of a flare of joint involvement (episodes of peripheral arthritis and dactylitis of the small joints of hands and feet once per week) and skin involvement, he was started on methotrexate 10 mg weekly with folate supplementation, in combination with cyclosporine 200 mg daily, with reduced extension and severity of skin lesions and decreased frequency of arthritis flares to once per month. However, he did not reach complete remission. Shortly after initiation, due to a 2-fold increase of liver enzymes (hepatitis excluded based on abdomen ultrasound and hepatitis B and C viral profile), methotrexate was reduced to 7.5 mg weekly [12]. The reduced methotrexate regimen led to normalization of the liver enzymes but was ineffective in controlling both skin and joint involvement. Two years later, due to elevated arterial pressure values (up to 150/90 mmHg) and serum creatinine increase >30% of baseline value (up to 1.4 g/L), cyclosporine was stopped. 

**Figure 2:**
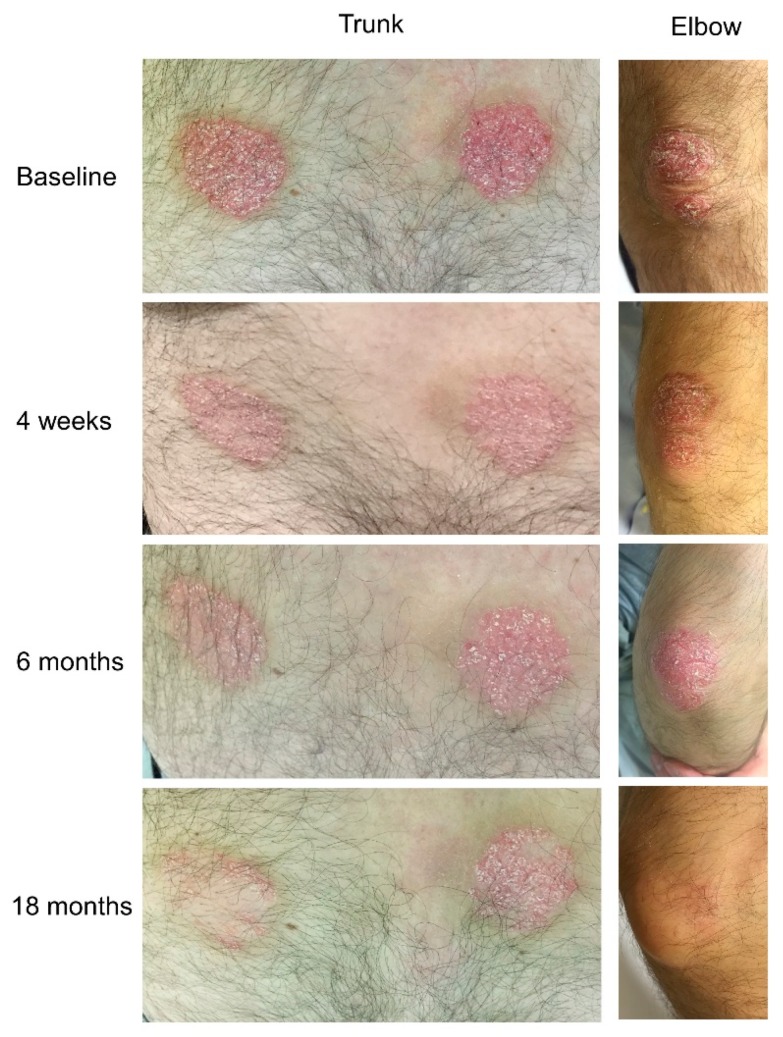
Psoriatic lesions of the trunk (**left**) and of the right elbow (**right**) at baseline and after 4 weeks, 6 months and 18 months of treatment with apremilast in combination with methotrexate. In the case of the right upper limb, the lesion has completely healed.

The following year, he referred to our outpatient clinic for skin and joint flare. At referral, physical examination demonstrated mild psoriasis of trunk, arms and legs, with a psoriasis area severity index (PASI) score of 5.1 (Figure 2), dactylitis of the third digit of the left foot and arthritis of the third interphalangeal joint of the left hand, which, together with a normal C reactive protein (CRP), activity visual analogue scale (VAS) and pain VAS of 4 and 4.5 respectively, accounted for a disease activity in psoriatic arthritis (DAPSA) score of 12 (low disease activity) [13]. 

**Figure 3:**
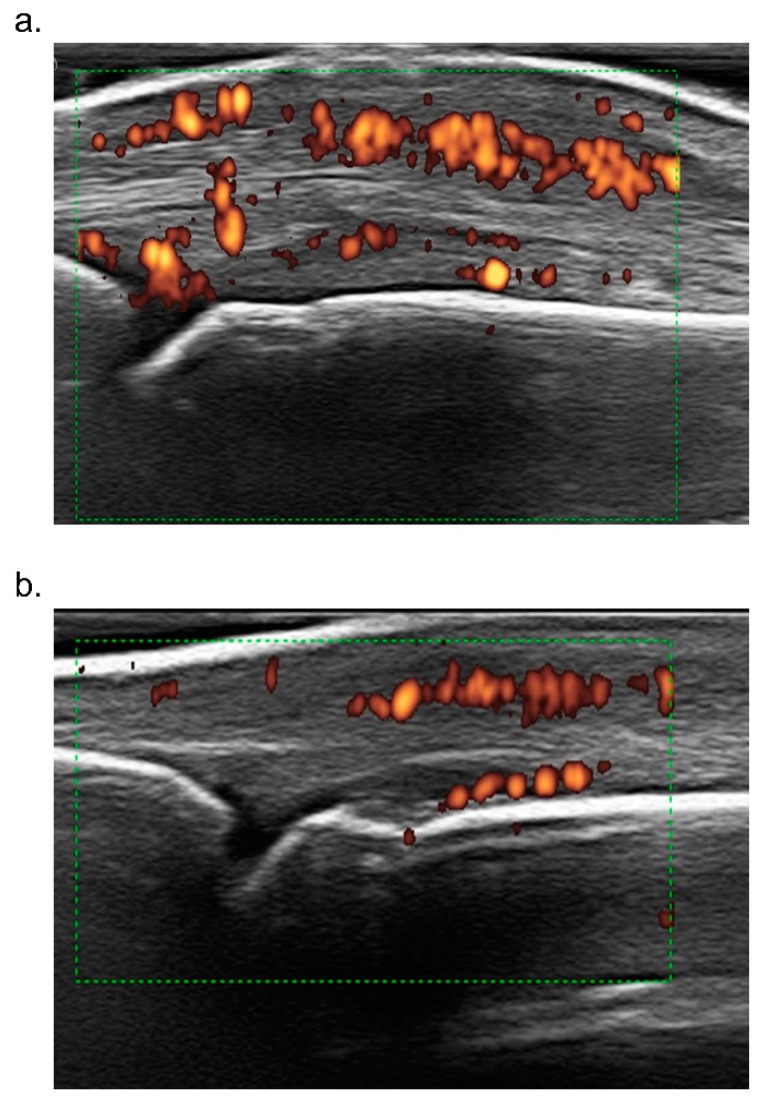
Ultrasound dorsal scan of the third metacarpophalangeal joint of the right hand of our patient. Swelling of the periarticular tissues, power doppler signal (red dots) of the joint and in the peritendon of the extensor of the finger were more evident at baseline (**a**). After 6 months, the power doppler signal and the periarticular swelling were considerably reduced (**b**).

Laboratory tests showed no relevant alterations. Radiographic assessment of hands and feet showed no erosions and no signs of axial involvement were found at magnetic resonance imaging (MRI). Ultrasound imaging of the third metacarpophalangeal (MCP) joint showed inflammation of the joint and of the peritendon of the extensor tendon (Figure 3a).

**Figure 4:**
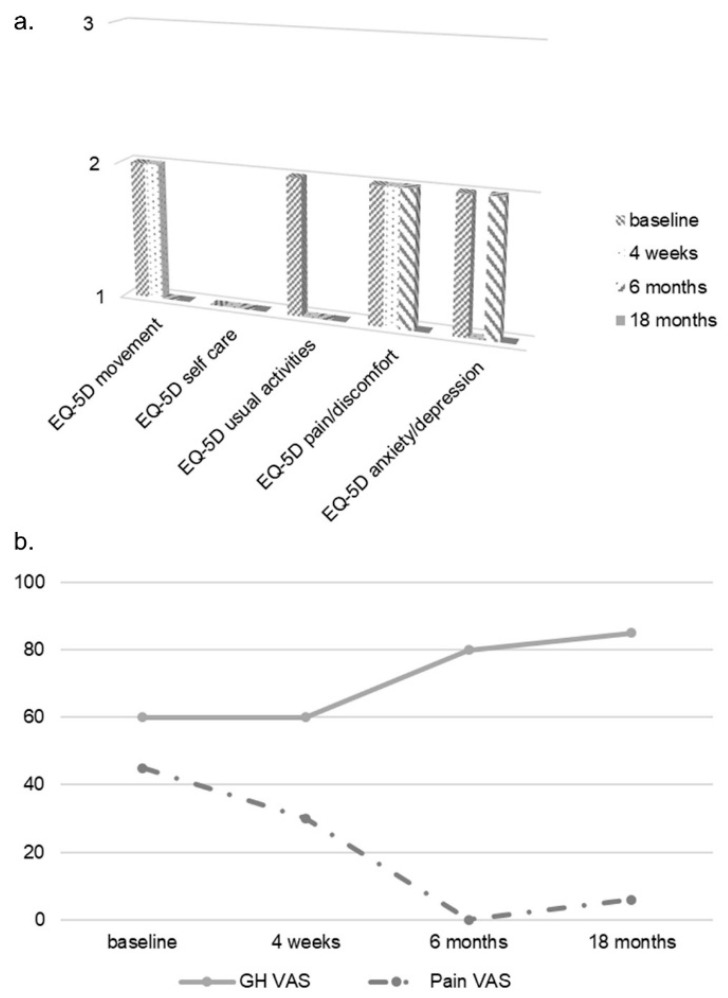
Health-related quality of life of the patient. The figure shows the results for each domain of EQ-5D (**a**), general health (GH) visual analogue scale (VAS) and pain VAS reported by the patient at baseline and during follow-up (**b**).

Considering that the patient failed to respond to two DMARDs (cyclosporine and methotrexate), that he refused a parenteral drug, that he presented mild skin and joint involvement, no bone erosions, dactylitis and no axial involvement, apremilast was chosen [4,5,6]. At baseline, the EQ-5D questionnaire reported altered HRQoL, with some difficulties in motility, no difficulties in self-care and some difficulties in usual activities, moderate pain/discomfort and moderately anxious/depressed mood. Patient’s global health (GH) VAS was 60 and pain VAS was 45 (Figure 4). Furthermore, the patient was screened at baseline for CV risk; carotid arterial doppler ultrasound demonstrated only intimal thickening of the left bulb and 24 h blood pressure monitoring demonstrated mild systolic and diastolic daily hypertension for which he started lercanidipine 10 mg/daily. At baseline, total cholesterol (TC) and triglyceride (TG) serum levels were 173 mg/dL and 113 mg/dL respectively, and TC/high density lipoprotein (HDL) ratio was 4.94 (Table 1). After 4 weeks, the extension and severity of erythema and infiltration of psoriatic plaques were substantially reduced (PASI 2.5, Figure 2). Furthermore, the patient reported a marked improvement of joint involvement with no further episodes of arthritis or dactylitis, although inflammatory morning stiffness persisted (DAPSA 6.0). At the 2-month follow-up, the patient reported several side effects such as mild headache, dizziness and hypotension and, due to further amelioration of the skin and joint involvement he stopped apremilast, which was restarted shortly after, due to worsening of psoriasis (PASI 3.0) and occurrence of a new joint flare (DAPSA 10). At the time he was still taking methotrexate 7.5 mg weekly. At the 6-month follow-up he reported no arthritis flares and no inflammatory morning stiffness. At physical examination, the third MCP joint was swollen but not tender (DAPSA 3), and ultrasound imaging showed reduction of joint and soft tissue inflammation (Figure 3b). A further improvement of skin involvement was observed and reported by the patient (PASI 1.2, Figure 2). During the follow-up, we observed an amelioration of the serum lipid profile already after 4 weeks, with a 5.8% reduction of TC and a reduction of TC/HDL ratio, and after 12 months, a further reduction of TC, LDL and TG values of 15.6%, 25.7% and 17.7% respectively, and a 20% increase of HDL levels compared to baseline values. TC/HDL ratio reached the favorable value of 3.48, as shown in Table 1. We even observed a 5% weight loss—although our patient already had a normal baseline weight and body mass index (BMI)—reaching a final normal weight with a normal body mass index (BMI 21.5). After 12 months, the clinical manifestations were stable. We therefore decided to stop methotrexate, but the patient experienced a slight relapse of both skin and arthritis (PASI 2.5, DAPSA 6), shortly after. Therefore, although dosage was low (7.5 mg), we reintroduced methotrexate, leading at the 18-month follow-up to stable minimal disease activity with PASI 75 and joint remission (PASI 1.2, DAPSA 2) [11,13] and a dramatic improvement in HRQoL (Figure 4).

## 3. Discussion

Apremilast was quickly effective in controlling mild skin and joint involvement, including dactylitis, and led to HRQoL improvement, in line with previous literature [7,8,9,14]. Side effects in our patient were mild and temporary and no laboratory pre-screening or ongoing monitoring were necessary, due to the limited organ toxicity [7].

PsA is associated with a higher prevalence of metabolic syndrome, obesity, diabetes mellitus and abnormal serum lipid levels, in particular hypertriglyceridemia, when compared to rheumatoid arthritis (RA) patients [15,16,17]. Our patient showed a baseline unfavorable TC/HDL ratio, very close to the proposed cut-off of >5 for high risk of CV events, although TC and TG serum levels were still in the normal range [18,19]. It is common knowledge that PsA is associated with a high prevalence of metabolic syndrome, obesity, diabetes mellitus and abnormal serum lipid levels, specifically hypertriglyceridemia [17,18,19]. Metabolic syndrome and psoriasis share a chronic inflammatory state, characterized by production of proinflammatory cytokines such as tumor necrosis factor-alpha (TNF-α) and interleukin (IL)-6 [20]. In a study comparing RA and PsA patients from the Consortium of Rheumatology Researchers of North America (CORRONA) registry, a higher proportion of patients in the PsA than in the RA group was found to have significantly higher mean TG serum levels, hypertriglyceridemia, and lower HDL serum levels with all these findings not correlated with the BMI of patients [17]. The higher prevalence of metabolic syndrome in PsA compared to RA patients could be explained by the presence of both skin and joint inflammation in patients with PsA, although different pathogenic mechanisms in the two diseases are likely involved [15,16,17]. Since the first month of treatment with apremilast, we observed a gradual and consistent TC, LDL, TG and TC/HDL ratio reduction, along with an increase in HDL serum levels.

**Figure 5:**
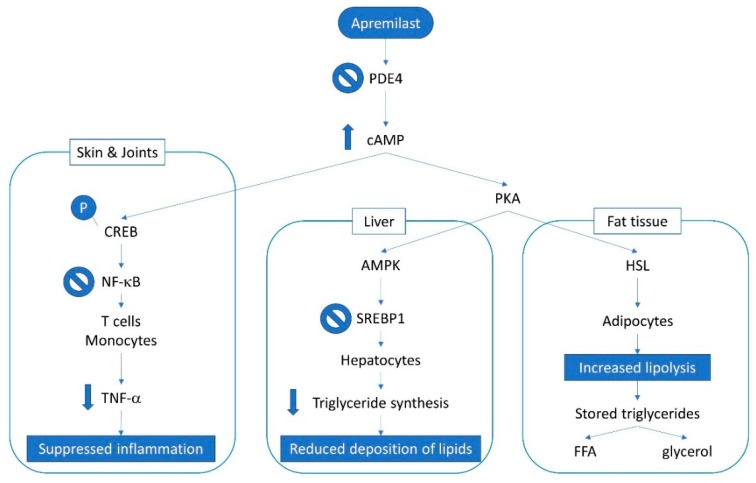
Overview of apremilast mechanism of action in psoriatic arthritis in different systems. Definitions: adenosine monophosphate kinase (AMPK); cyclic adenosine monophosphate (cAMP); cAMP response element-binding (protein) (CREB); free fatty acids (FFA); hormone-sensitive lipase (HSL); nuclear factor κB (NF-κB); phosphodiesterase 4 (PDE4); phosphokinase A (PKA); sterol regulatory element-binding protein 1 (SREBP1); tumor necrosis factor-alpha (TNF-α).

Apremilast is a tsDMARD directed against PDE4 that works by blocking the degradation of cyclic adenosine 3′,5′-monophosphate (cAMP) to adenosine 3′,5′-monophosphate AMP, thus increasing intracellular cAMP levels, which in turn activate protein kinase A (PKA) leading to the phosphorylation of cAMP response element binding protein. This eventually leads to the inactivation of nuclear factor κB (NF-κB) activity in plasmacytoid dendritic cells and T-cells, and the reduction of Th1-derived synthesis of pro-inflammatory cytokines (Figure 5) [21]. PDE4 also acts as a critical regulator of metabolic processes [22,23,24,25]. In the liver, the increase in cAMP levels promotes AMP kinase (AMPK) activity, thus inhibiting the accumulation of lipids via the regulation of lipid metabolism-related factors, such as sterol regulatory element-binding protein 1 (SREBP1) [26]. Non-alcoholic fatty liver disease (NAFLD) is strongly associated with metabolic syndrome [27] and both NAFLD and metabolic syndrome have a high prevalence in psoriasis and in particular in those patients who also have PsA [28,29]. Intriguingly, PDE4 inhibition has been demonstrated to have an anti-inflammatory effect also in non-alcoholic steatohepatitis (NASH) [30].

In fat tissue, the raise in cAMP levels induced by cAMP-PKA phosphorylation activates hormone-sensitive lipase (HSL), thus increasing lipolysis, the process responsible for the hydrolysis of the stored TG to free fatty acids (FFA) and glycerol (Figure 5) [25,31]. Our patient, who already had a normal BMI, also experienced a slight loss of weight only partly due to mild gastrointestinal side effects, which resolved after 6 months of treatment. However, he did not report any diet modification.

Cholesterol efflux capacity (CEC) is the ability of HDL to remove cholesterol out of macrophage foam cells and is impaired in atherosclerosis [32]. A very recent study demonstrated a reduced CEC in patients with NAFLD, thus providing a proof of concept of the link between NAFLD and the development of atherosclerosis. Notably, there is evidence that PDE4 inhibition may lead to increases in ATP cassette binding protein 1 (ABCA1) expression and cholesterol efflux mediated by apolipoprotein A-I, which is responsible for the removal of excess cholesterol from macrophage foam cells in the arterial wall [33].

These mechanisms may have led in our patient to the reduction of TG serum levels and the normalization of TC/HDL ratio. Although our data need to be confirmed with further follow-up and observational studies, these effects may contribute to a reduction of the development and progression of dyslipidemia, obesity and steatohepatitis in patients with PsA, possibly reducing CV risk in these patients [1,25].

In our case, apremilast was more effective when co-administered with methotrexate than alone. This observation could be explained by the fact that although both drugs increase intracellular cAMP, they exert different mechanisms of action: apremilast inhibits PDE4-mediated cAMP degradation; methotrexate increases adenosine levels, in particular via the activation of the adenosine 2A receptor [34]. The safety of this combination has already been demonstrated by pharmacokinetic studies [35].

Finally, HRQoL improved considerably and constantly during the 18-month follow-up (Figure 4). This is an important issue because the patient reported the reduction in HRQoL to be one of the main complaints at referral and it is consistently one of the main characteristics of PsA [1].

## 4. Conclusions

To the best of our knowledge, this is the first report of a possible metabolic effect of apremilast on serum lipid profile in a patient with PsA treated for mild skin and joint involvement. Future studies may be useful to better define the use of apremilast in patients with PsA and metabolic alterations.

## Figures and Tables

**Table 1:** Serum lipid profile of the patient at baseline and during follow-up after the introduction of apremilast. The percentage of reduction/increase of the serum levels are reported.

	Baseline	4 Weeks	6 Months	12 Months
TC mg/dL (% reduction)	173	163 (5.8)	153 (11.6)	146 (15.6)
HDL mg/dL (% increase)	35	34 (−2.86)	45 (28.6)	42 (20)
LDL mg/dL (% reduction)	115	101 (12.2)	98 (14.8)	85.4 (25.7)
TG mg/dL (% reduction)	113	118 (−4.4)	50 (55.7)	93 (17.7)
TC/HDL ratio	4.94	4.79	3.40	3.48

TC total cholesterol, HDL high density lipoprotein, LDL low density lipoprotein, TG triglyceride.
